# A case of malignant transformation of a serous borderline ovarian tumor effectively treated with BRAF/MEK inhibitor combination

**DOI:** 10.1016/j.gore.2024.101417

**Published:** 2024-05-16

**Authors:** Manrose Singh, Samantha Cornwell, Ariel Shaddaie, Leah Wachsmuth, Ashwin Ragupathi, Leonidas Salichos, Sandra Nissel-Horowitz, Rajasree Roy, Maria Plummer, Dong Zhang, Bhoomi Mehrotra

**Affiliations:** a Department of Biomedical Sciences, College of Osteopathic Medicine, New York Institute of Technology, Old Westbury, NY 11568, USA; b Center for Cancer Research, New York Institute of Technology, Old Westbury, NY 11568, USA; c Catholic Health Cancer Institute at St. Francis Hospital & Heart Center, East Hills, NY 11548, USA

**Keywords:** SBOT, Adenocarcinoma, Müllerian, BRAF

## Abstract

We describe a patient diagnosed with a metastatic adenocarcinoma of Müllerian origin, harboring a BRAF V600E mutation, ten years after being treated for a serous borderline tumor (SBOT). While BRAF mutations in the setting of SBOTs are common, they have been typically associated with a low chance of transformation or recurrence. The therapeutic approach, which combined hormone inhibition with receptor tyrosine kinase inhibitors (dabrafenib and trametinib), has demonstrated notable and enduring efficacy. This is clinically evidenced through serial PET-CT scans with sustained responses and extended progression-free survival, and serologically confirmed by monitoring CA-125 levels. This case demonstrates the critical role of early next-generation sequencing in detecting actionable molecular changes in rare cancers and possible metastases. It provides valuable insights into treating uncommon Müllerian adenocarcinomas and underscores the importance of targeted therapies in achieving long-lasting treatment outcomes.

## Highlights

• In 2008, a female patient was diagnosed with serous borderline ovarian tumor (SBOT)
• The patient was diagnosed with adenocarcinoma of Mullerian origin in 2018.
• Tumor profiling of the patient’s adenocarcinoma via Next Generation Sequencing identified the BRAF^V600E^ mutation in 2020.
• The patient has been treated with dabrafenib and trametinib since 2020 and remains progression-free.

## 1 Introduction

Adenocarcinomas originating from Müllerian tissues represent a heterogeneous cohort of gynecological malignancies, including ovarian, fallopian tube, and peritoneal cancers (Cobb et al., 2015). Among these cancers, ovarian cancer ranks first in lethality and third most common among affected populations (Liberto et al., 2022). A combination of wide molecular heterogeneity and a lack of effective early screening tools contributes to the high mortality associated with these cancers (Liberto et al., 2022). Most ovarian tumors are of epithelial origin and among this group, serous tumors are the most common type. Serous tumors are further classified into several subtypes, including the serous borderline ovarian tumors (SBOT), low-grade serous carcinomas (LGSC), and high-grade serous carcinomas (HGSC) (Kurman & Shih, 2016).

SBOTs have generally favorable prognoses, even in the presence of lymph node involvement (Qian et al., 2018). These tumors share several features with the LGSCs and harbor latent transformation potential, serving as precursor lesions for LGSCs (Longacre et al., 2005). The transformation into LGSC is associated with significantly poorer patient outcomes;thus, long-term monitoring of these patients is critical (Longacre et al., 2005). One of the shared features among SBOTs is the presence of activating mutations in the mitogen-activated protein kinase (MAPK) pathway, most often exclusively KRAS or BRAF (Kurman & Shih, 2016). Intriguingly, BRAF mutations are thought to have a protective effect against further transformation into LGSCs (Malpica and Wong, 2016, Tsang et al., 2013). These driver mutations can be a double-edged sword: they boost cell proliferation but also offer a clinical target for certain FDA-approved inhibitors (Zaman et al., 2019).

Here, we report on a patient with a history of a serous borderline ovarian tumor that recurred into a widespread, metastatic adenocarcinoma with Müllerian pathology almost ten years after undergoing a total abdominal hysterectomy and bilateral salpingo-oophorectomy (TAHBSO). This patient was found to have a BRAF^V600E^ mutation that was extremely responsive to receptor tyrosine kinase inhibitor treatment targeting BRAF and MEK (dabrafenib and trametinib, respectively).

## 2 Case presentation

In March 2018, a 63-year-old female presented to the emergency department with an initial complaint of chest pain. Her past medical and surgical histories were notable for a prior SBOT diagnosed in 2008, involving both ovaries. She was previously treated with a TAHBSO and lymph node resection. Her tumor was reported to be 7.0 cm in size (at its greatest dimension) with non-invasive desmoplastic implants on both ovaries, the left fallopian tube, and the left upper peritoneum. It involved the left and right pelvic lymph nodes and the *para*-aortic lymph nodes. There was microinvasion of the right ovary. Per pathology, the tumor was reported to be well-differentiated and TNM staging was given as T2c N1 Mx. Her family history was notable for breast cancer in her mother (type unknown) and lobular carcinoma in situ and intraductal papilloma in her sister.

**Fig. 1:**
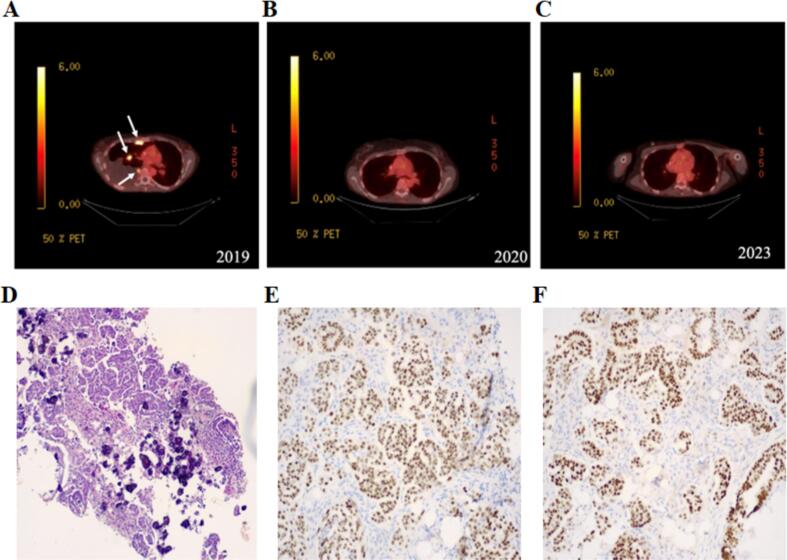
(A) PET-CT imaging showing significantly abnormal FDG uptake (indicated by white arrows) in metastatic regions in anterior chest wall and lung parenchyma. (B) The same region following six months of dabrafenib and trametinib treatment followed by (C) three years of combination treatment with dabrafenib and anastrozole, with no abnormal FGD uptake in the same region. (D) Histopathology showing H&E staining of nodule obtained from the anterior chest wall. Panel (E) indicates positive staining for ER and panel (F) shows staining for PAX8.

For evaluation of her new onset chest pain, she underwent a cardiology assessment which was unremarkable. Imaging of the chest revealed metastases to the chest wall and lung. Her serum CA-125 was elevated at 285 U/mL **(**Fig. 3**B).** PET-CT scan revealed abnormal FDG uptake in areas in the right pleural chest wall regions as well as in the abdominal, retroperitoneal, and pelvic lymph nodes (Fig. 1**A**). Given the concern for potential malignancy, the patient underwent multiple CT-guided needle core biopsies. Pathology of biopsy specimens demonstrated malignant cells consistent with adenocarcinoma with multiple areas of extensive calcifications **(**Fig. 1**D).** Direct comparison with slides from her prior ovarian tumor showed similar histological findings, leading to a concern for recurrence with metastatic spread as the specimens were of Müllerian origin. Immunohistochemistry staining of the tumor indicated it was positive for PAX-8, estrogen receptor, CK7, and WT-1 and negative for CK20, TTF1, napsin A, and villin (Fig. 1**E and** Fig. 1**F)**. Combination of PAX8, ER, WT-1, CK7, and CK20 findings favored a tumor of serous Müllerian origin. Per pathology, diagnosis was reported as “adenocarcinoma of Müllerian origin”.

**Fig. 2:**
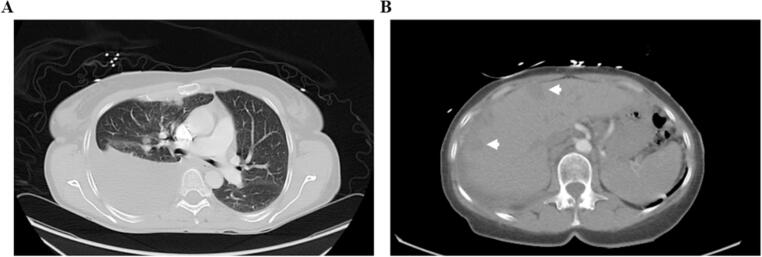
CT imaging of malignancy from March 2019 showing disease progression. (A) Section of lung parenchyma with large right-sided pleural effusion and calcified metastatic nodules. (B) Section of liver with likely hepatic metastases, indicated by arrows.

**Fig. 3:**
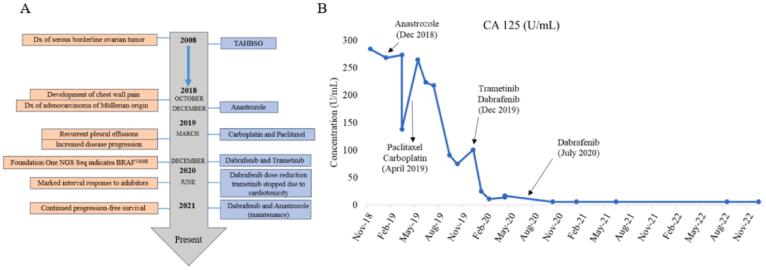
(A) Timeline of patient disease and treatment course, with disease progression on left and treatments on right. (B) Graphical depiction of CA-125 levels over patient treatment course.

Given ER positivity, the patient was started on anastrozole. After 4 months of mild improvement, she developed a recurrent right-sided pleural effusion. Cytology confirmed adenocarcinoma (Fig. 2**A)**. Interval CT imaging at the time also revealed progression with additional sites of probable metastases in the liver **(**Fig. 2**B)**. Paradoxically, the CA-125 levels at this time declined significantly for a short period of time even with clinical and radiographic disease progression (Fig. 3**B**).

She was administered four cycles of chemotherapy with carboplatin and paclitaxel, followed by resumption of anastrozole. Tumor profiling via NGS at FoundationOne was notable for a BRAF^V600E^ mutation, MYC, and EPHB1 amplifications, as well losses in CDNK2A, CDNK2B, and MTP. Notably, p53, BRCA1, and BRCA2 were wild-type. Given the presence of the actionable BRAF^V600E^ mutation, the patient was started on combination dabrafenib and trametinib therapy. Follow up PET-CT scans showed significant improvement in the previously abnormal FDG positive metastatic chest and lung areas within 6 months of treatment **(**Fig. 1**B and** Fig. 1**C).** Serum CA-125 levels continued to decline throughout the rest of her treatment **(**Fig. 3**B).** After eight months of treatment of dual-inhibition therapy, the patient was transitioned to a lower dose of single-agent dabrafenib regimen due to the cardiotoxicity of the combination regimen. She remains on a maintenance regimen of dabrafenib and anastrozole at the time of this publication with no indication of tumor progression (Fig. 1**C**). Timeline of patient diagnosis and treatment course is presented in Fig. 3**A**.

## 3 Discussion

We report the unique case of a patient who previously had an SBOT and is now, ten years later, diagnosed with an adenocarcinoma of Müllerian origin associated with a BRAF V600E mutation. Histological comparison between the patient's new adenocarcinoma and the previous tumor exhibited strikingly similar features, with the metastases displaying an augmented presence of calcifications. Initial treatment of the adenocarcinoma included debulking efforts with chemotherapy and estrogen-receptor inhibition. Given the widespread nature of the disease and location of the metastases in the pleura, surgical resection was not undertaken in this case. Sequencing revealed a BRAF^V600E^ mutation, which is common in subsets of LGSC tumors and several other cancer types (Kurman & Shih, 2016). The targeted co-inhibition of BRAF and MEK resulted in a sustained tumor response as shown in the PET-CT scans, ongoing progression free survival and the normalization of serum CA-125 levels. The high degree of success of this treatment is further evidence that NGS sequencing should be obtained as early as possible to identify actionable molecular alterations in guiding treatments for uncommon cancers.

The SBOT is a tumor type with a generally good prognosis but can transform into a low-grade or high-grade serous carcinoma. Questions remain regarding the pathways of malignant transformation that took place in this patient and the role of BRAF^V600E^ mutation. A significant caveat in this case is that we were unable to sequence the original SBOT and determine if the BRAF mutation was present. Considering the patient's comprehensive medical history and the findings from histopathological studies and immunohistochemical tests, the chest wall lesion identified in 2018 as “adenocarcinoma of Müllerian origin” most likely represents metastatic growth from the initial ovarian tumor diagnosed in 2008 as an SBOT. This recurrence as a metastatic tumor is due to the transformation of the original tumor, exacerbated by lymph node involvement and the presence of microinvasive disease noted in 2008. Müllerian epithelial cancers encompass a group of epithelial cancers traditionally known as ovarian carcinomas, and extend to include cancers from Müllerian duct-derived organs such as the uterus and cervix (Cobb et al., 2015, Dubeau, 2015).

Intriguingly however, several studies actually show that the presence of the BRAF mutation in an SBOT is associated with a decreased chance of progression to serous carcinoma (Malpica and Wong, 2016, Chui et al., 2019). In one study, SBOTs with BRAF mutations showed decreased micropapillary variant histology, were more frequently stage I and had a lower prevalence of endosalpingiosis (Chui et al., 2019). BRAF mutations have also been linked to features suggestive of cellular senescence, suggesting a lower risk of transformation (Seidman et al., 2020). On the other hand, KRAS mutants were found to more likely undergo malignant transformation when compared to the BRAF counterparts in SBOTs (Tsang et al., 2013). This case serves as an important counterexample to these findings, underlining the significance of somatic testing to detect molecular alterations that can guide the treatment of rare cancers.

Other risk factors for the transformation of SBOTs into malignant carcinomas include bilateral ovarian disease, microinvasion, ovarian surface involvement, advanced disease stage, and invasive implants (Grisham et al., 2023, Vang et al., 2017). Among these, our patient previously had bilateral ovarian disease and right ovary microinvasion. According to Grisham et al., non-invasive implants, such as those our patient had, increase the risk of progressing to LGSC by 15–20%. However, the absence of invasive implants and other aggressive disease markers suggested a less aggressive disease trajectory (Grisham et al., 2023). Their analysis also noted that patients with LGSC tend to show varying responses to MEK inhibitors depending on the MAPK alteration status (Grisham et al., 2023). Additionally, some lymph node involvement was noted in our patient, but there is no established link to indicate a more aggressive disease course (Longacre et al., 2005). Therefore, further research is needed to determine the degree to which these risk factors contribute to metastatic transformation and to consider the role of other variables like patient age and surgical extent. Consequently, thorough histopathological and molecular evaluations are crucial for assessing risk and managing SBOTs' progression to serous carcinomas.

The efficacy of treatment in this patient relied on successful MAPK pathway inhibition. The MAPK pathway, consisting of a hierarchical arrangement of signaling proteins, is one of the most well-characterized oncogenic signal transduction pathways and is reviewed in detail elsewhere (Braicu et al., 2019). Importantly, constitutive expression of BRAF mutants has been linked to the sustained activation of several key transcription factors and ultimately, the increased cell proliferation and growth of cancer cells (Braicu et al., 2019). Among these mutants, the V600E mutation is the most common and studied. From a sample size of 2,983 serous ovarian tumors, Campos and colleagues noted an overall BRAF^V600E^ mutation rate of 1.7%. However, this subset only included 15 SBOTs, of which 13.3% contained this mutation (Campos et al., 2018).

While BRAF inhibitors have shown efficacy in multiple cancers, many patients eventually develop resistance, contributing to the dysregulation of cell proliferation and differentiation. To overcome potential resistance, several combinations targeting multiple MAPK-proteins in addition to BRAF/MEK are being explored as treatment options. A recent clinical trial demonstrated the potential of combining avutometinib (MEK inhibitor) and defactinib (FAK inhibitor) in targeting multiple proteins within the MAPK pathway, showing durable responses in recurrent LGSC (Banerjee et al., 2023). MAPK inhibitor as monotherapies also represent an important clinical avenue to consider. Selumetinib, a MEK inhibitor, provided considerable disease control in recurrent LGSC, supporting the use of targeted therapy approaches (Farley et al., 2013). Gershenson et al. studied the efficacy of trametinib against standard chemotherapy options, finding that trametinib significantly improved progression-free survival, highlighting its potential as a new standard of care for LGSC (Gershenson et al., 2022).

Several recent case reports identified this BRAF/MEK combination having dramatic clinical outcomes for ovarian cancer patients (Mendivil et al,. 2018, Lima et. al, 2022). In addition, Hendriske and colleagues performed a meta-analysis on the use of MAPK inhibitors in LGSC, and found that combined treatments with RAF and MEK inhibitors had the highest efficacy in V600E positive cancers (Hendriske et. al, 2023). However, specific evidence for the successful use of the dual MAPK therapy for transformed serous borderline ovarian tumors is rare, and here we are among the first to describe a sustained tumor response with BRAF, MEK, and ER inhibition. The sustained response to BRAF-directed therapy, three years after initiation, is a notable achievement in this unique disease setting. The overall success of the targeted therapy as compared with single modalities such as hormonal or chemotherapy alone suggests this as a viable treatment option for selected patients.

## 4 Consent statement

The manuscript is carefully reviewed to avoid patient identification details and/or figures. Written consent has been obtained from the patient and is held on file at our institution. A copy of the written consent is available for review by the Editor-in-Chief of this journal on request.

## CRediT authorship contribution statement

**Manrose Singh:** Writing – original draft, Data curation. **Samantha Cornwell:** Writing – review & editing. **Ariel Shaddaie:** Writing – review & editing. **Leah Wachsmuth:** Writing – review & editing. **Ashwin Ragupathi:** Writing – review & editing. **Leonidas Salichos:** Writing – review & editing. **Sandra Nissel-Horowitz:** Data curation. **Rajasree Roy:** Data curation. **Maria Plummer:** Writing – review & editing, Supervision. **Dong Zhang:** Writing – review & editing, Supervision. **Bhoomi Mehrotra:** Writing – review & editing, Supervision, Conceptualization.

## Declaration of competing interest

The authors declare that they have no known competing financial interests or personal relationships that could have appeared to influence the work reported in this paper.
